# Neuropathology

**Published:** 1891-09

**Authors:** 


### Selections and Abstracts

### NEUROPATHOLOGY.

THE RELATION OF EYE-STRAIN TO GENERAL MEDICINE.—George M. Gould (Med. News, Aug. 23, 1890), in an eminently practical address before the Philadelphia Hospital Medical Society, says that eye-strain is an enormously frequent, fertile and unsuspected source of non-ocular disease. The author's remarks are of especial interest, as his observations were made in his own practice. He asks:

1. What is eye-strain?
2. What is it's etiology?
3. How is it diagnosed?
4. What are it's effects?

The term eye-strain is usually applied to the irritational inco-ordinations and abnormal exertions of the intra-ocular or extra-ocular muscles. The simple muscular use or strain is, if not entirely absent, often but a small factor. The ciliary muscle is more overworked than the muscles of the hand, whilst the effects are dissimilar. The muscular element, *per se*, is subordinate and *post hoc*. Failure to see this point has led to the woeful error of those extremists who cure all the ills that eyes are heir to by tenotomies. In the greatest eye-strains patients will aver that the eyes give no trouble. Insufficiency is a mere consequence of ametropia. Correct this and the muscle equilibrium is generally restored. Tenotomy before a lengthy trial of this procedure, is no less than surgical barbarism. The insufficiency is a phenomenon of nerve centers and nerve forces, rather than of strengths or strains of muscle fibre. The etiology of much insufficiency is the disturbance produced by ametrophia of the normal relation of accommodation and convergence. *This is patently a nervous phenomenon, pure and simple.* [Italics ours.] This ametropia is almost limited to two varieties, hyperopia and astigmatism. In myopia generally there can be no strain, for there is already too much refraction, hence there can be no strain.

The author believes hyperopia and astigmatism are more common now than ever before. Printing, sewing, schools, commercialism, have given the eye tasks for which it was never made. When eye-strain is fully recognized, spectacles will be as common as noses. The greatest good of spectacles will be prophylactic. The day will come when a conscientious parent will never permit a child, however apparently healthy, to grow to puberty without a scientific examination of the eyes having been made and repeated at intervals thereafter. The consequences of eye-strain are so subtile, so varied, so remote, so peculiar, so unforeseen and so harmful, that only by this plan can they be obviated. A farmer can safely carry for a lifetime five or ten times the defect that a city girl could endure for only a day.

Headache from eye-strain may be recognized by these points: 1. The patient is generally a girl or woman; 2. Pain is located in forehead or temples; 3. Near work aggravates it; 4. It is frequently associated with digestive disorders.

The author concludes by charging the common school system with many of the troubles he has described.—*T. D.* in *Alienist and Neurologist.*

